# Mechanical thrombectomy: Lessons to be learned from intravenous thrombolysis

**DOI:** 10.1002/brb3.1500

**Published:** 2019-12-13

**Authors:** Rajiv Advani, Soffien Ajmi, Martin W. Kurz

**Affiliations:** ^1^ Stroke Unit Department of Neurology Oslo University Hospital Oslo Norway; ^2^ Neuroscience Research Group Stavanger University Hospital Stavanger Norway; ^3^ Department of Neurology Stavanger University Hospital Stavanger Norway; ^4^ Faculty of Health Sciences University of Stavanger Stavanger Norway; ^5^ Institute of Clinical Medicine University of Bergen Bergen Norway

Intravenous thrombolysis (IVT) was approved for the treatment of acute ischemic stroke (AIS) in 1995 (Hacke et al., 1995). The treatment window was subsequently expanded from 3 to 4.5 hr after symptom onset in 2008 (Hacke et al., 2008). The initial implementation of IVT administration in the expanded therapeutic window was troublesome, and only, very few AIS patients received IVT. Treatment rates as low as 3% were reported across Europe 10 years after the approval of IVT for AIS (Leys et al., 2009). Despite this, IVT treatment rates within individual countries varied immensely, ranging from 5% to 20% (Wijngaarden et al., 2009). The differing organizational systems at individual hospitals were the most decisive factor.

This led some treatment centers to begin restructuring their organizational systems (Advani, Naess, & Kurz, 2014; Meretoja et al., 2012). These changes led to significant improvements in the numbers of patients receiving treatment and in conjunction with informational campaigns unprecedented IVT treatment rates, consistently above 30%, and in periods approaching 50% were seen (Advani, Naess, & Kurz, 2016). High rates of IVT treatment were seen to be, at least in part, a by‐product of lowering in‐hospital treatment times. However, the reduction of delay in the prehospital arena through the restructuring of triage has played a pivotal role (Patel, Rose, O'Brien, & Rosamond, 2011).

These improved door‐to‐needle times, a result of systematic organizational changes, led not only to improved treatment rates, but also improved clinical outcomes (Advani, Naess, & Kurz, 2017; Meretoja et al., 2014). There seems to be a link between the number of thrombolysed patients at a treatment center and a lower mean door‐to‐needle time (Bray et al., 2013). This relationship has been dubbed the “Bigger, Faster?” link, but can also be seen as a practice‐makes‐perfect metaphor.

It is important to note that these higher IVT treatment rates have only been achieved at certain stroke treatment centers and the majority of AIS patients are admitted to centers with lower treatment rates (Reuter et al., 2017). This means that many AIS patients are precluded from treatment owing to the lack of implemented organizational changes. As many works have shown, the most common reason for IVT ineligibility is delay from onset (Reiff & Michel, 2017).

From 2015 onwards, the use of mechanical thrombectomy (MT) was approved for the treatment of AIS caused by large vessel occlusion (LVO; Goyal, Menon, et al., 2016). The widely accepted time frame for the treatment of these patients is up to 6 hr after the onset of symptoms. However, newer MT trials are already pushing those boundaries and extending time frames up to 16 hr (Albers et al., 2018) and 24 hr (Nogueira et al., 2018) after symptom onset. Despite the extended time frames for MT, better clinical outcomes are seen where treatment is more rapidly expedited (Goyal, Hill, Saver, & Fisher, 2016). The impact of time‐saving on clinical outcome is even more pronounced in the setting of LVO treatment (Meretoja, Keshtkaran, Tatlisumak, Donnan, & Churilov, 2017).

As observed during the implementation phase of IVT treatment, the rates of MT seen during the initial years have been rather low. MT rates in the later treatment time frames, up to 16 and 24 hr, respectively, are even lower. This seems to be a case of history repeating itself.

As discussed above, the lack of implemented cultural, informational, and organizational changes at most treatment centers lay at the heart of the low IVT treatment rates. Systematic improvements at certain treatment centers led to an increase in IVT rates, in stark contrast to centers where these changes had not been implemented.

Mechanical thrombectomy is pivotal in the setting of LVO stroke for achieving a good clinical outcome; the number needed to treat is as low as 2.5 (Goyal, Menon, et al., 2016). The cost effectiveness of MT with or without adjuvant IVT is unquestionable, and this begs the question: how long will it take before the lessons from IVT implementation are learned? It is also worth noting that the changes made to organizational systems and their positive results were not bought about overnight. The methodical changes leading to the streamlining of treatment systems take persistence and endurance if sustainable results are to be achieved. A key point to be mentioned here is the availability of MT; in remote areas, longer transport times are involved and this has a significant effect on treatment outcome (Perez de la Ossa et al., 2016). This again brings the focus back around to expediting treatment as quickly as possible in all treatment centers.

With the goal being to expand the use of MT to as many eligible LVO stroke patients as possible, the implementation of changes should be expedited without unnecessary delay. In this setting, it is worthwhile mentioning the futility of prehospital LVO recognition scales (Anadani, Almallouhi, Wahlquist, Debenham, & Holmstedt, 2019). These scales were designed to triage patients suspected of having a LVO stroke to a comprehensive stroke treatment center where MT could be performed. However, these scales have a significant false‐negative and false‐positive values.

This implies the need for a combination of well‐documented organizational changes, informational campaigning alongside the implementation of more innovative concepts such as simulation training. Advances in simulation training have shown a reduction in door‐to‐needle time for IVT treatment to 13 min (Ajmi et al., 2019). Other solutions in telemedicine and prehospital stroke care systems seem to offer promise when it comes to improving treatment rates while improving onset to treatment times. A combination of tried and tested methods and innovative newer solutions seems to be the way forward in the pursuit of increased treatment rates not only for MT, but IVT as well. At centers where organizational and informational changes have not yet been implemented, the advent of a treatment modality as potent as MT should serve as a call to action.

The implementation of such systems is not only crucial for the clinical outcome of AIS and LVO stroke, but also has a greater perspective in the setting of healthcare economics. The changes that need to be made to increase MT rates lie in lessons being learned from the implementation of IVT.

## CONFLICT OF INTEREST

The authors report no conflicts of interest.

## Data Availability

Data sharing is not applicable to this article as no new data were created or analyzed in this study.
